# ITGA5 promotes tumor angiogenesis in cervical cancer

**DOI:** 10.1002/cam4.5873

**Published:** 2023-03-31

**Authors:** Xiaohan Xu, Lulu Shen, Wenhan Li, Xiaoli Liu, Ping Yang, Jing Cai

**Affiliations:** ^1^ Department of Obstetrics and Gynecology Union Hospital, Tongji Medical College, Huazhong University of Science and Technology Wuhan China; ^2^ Department of Obstetrics and Gynecology First Affiliated Hospital, School of Medicine, Shihezi University Shihezi China

**Keywords:** angiogenesis, cervical cancer, ITGA5, VEGFA

## Abstract

**Purpose:**

Integrins are critical to cancer progression. Integrin alpha 5 (ITGA5) is correlated with the prognosis of cervical cancer patients. However, whether ITGA5 plays an active role in cervical cancer progression or not remains unknown.

**Methods:**

ITGA5 protein expression was detected in 155 human cervical cancer tissues by immunohistochemistry. Data from The Cancer Genome Atlas were utilized to identify risk factors for the overall survival of cervical cancer patients and ITGA5‐associated differentially expressed genes. Analyses of single‐cell RNA‐seq based on Gene Expression Omnibus datasets were performed to show the coexpression of ITGA5 and angiogenesis factors. Tube formation assay, 3D spheroid sprout assay, qRT‐PCR, Western Blotting, ELISA, and immunofluorescence were conducted to explore the angiogenic function of ITGA5 *in vitro* and underlying mechanisms.

**Results:**

High ITGA5 level was significantly correlated with increased risk in terms of overall survival and advanced disease stage in cervical cancer patients. ITGA5‐associated differentially expressed genes linked ITGA5 to angiogenesis, and immunohistochemistry showed a positive correlation between ITGA5 and microvascular density in cervical cancer tissues. Moreover, tumor cells transfected with ITGA5‐targeting siRNA decreased ability to promote endothelial tube formation *in vitro*. ITGA5/VEGFA coexpression was observed in a tumor cell subpopulation and the decreased endothelial angiogenesis by downregulating ITGA5 could be reversed by VEGFA. Bioinformatics analysis highlighted the PI3K‐Akt signaling pathway as downstream of ITGA5. Downregulation of ITGA5 in tumor cells significantly decreased p‐AKT and VEGFA levels. Fibronectin (FN1) coated cells or transfected with FN1‐targeting siRNA showed fibronectin may play a critical role on ITGA5‐mediated angiogenesis.

**Conclusion:**

ITGA5 promotes angiogenesis and possibly be a potential predictive biomarker for poor survival of patients in cervical cancer.

## Graphical abstract

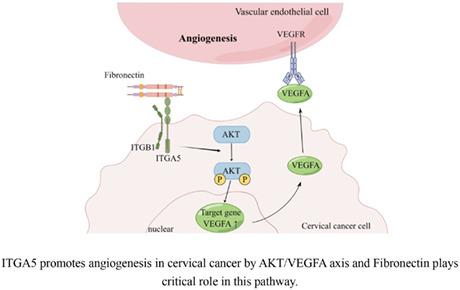

## 1 INTRODUCTION

Cervical cancer is the second most frequent diagnosed female genital system malignant tumor worldwide and ranks highly among cancers in the female population in terms of morbidity and mortality rates in the world.^1^, ^2^ Patients with early stages have a relatively favorable prognosis, while those with advanced cervical cancer usually have a poor clinical outcome.^3^ Hence, there remains a great challenge to improve the prognosis of patients with advanced and progressing disease.

Integrin family consists of 24 heterodimeric receptors that mediate adhesion to various extracellular matrix (ECM) components.^4^ As surface adhesion receptors, integrins also play a key role in signal transduction to regulate cytoskeletal organization, cell migration, proliferation, and survival.^5^ The function of integrins is regulated by a variety of mechanisms, including conformational changes, protein–protein interactions, and trafficking, and has been implicated in nearly every stage of cancer development, from initial tumor formation to extravasation at metastatic sites and metastatic niche formation.^6^, ^7^ By using bioinformatics methods, we comprehensively explored the correlations of 19 integrin superfamily members with the overall survival (OS) of cervical cancer patients and found that integrin alpha 5 (ITGA5) was the highest risk factor for prognosis.

ITGA5 mainly functions as a receptor of fibronectin in form of a heterodimer combined with integrin beta 1.^8^ ITGA5 is highly expressed in a panel of tumors and associated with poor survival in patients with laryngeal squamous cell carcinoma.^9^, ^10^ Functionally, ITGA5 has been reported to promote tumor progression, metastasis, and drug resistance. In gastrointestinal tumors, ITGA5 promoted tumor progression through the activation of the FAK/AKT pathway and was correlated with tumor purity and levels of immune infiltration.^11^, ^12^ ITGA5 in breast cancer was identified as a mediator and a therapeutic target of bone metastasis.^13^ In addition, ITGA5 has been found to mediate chemotherapy resistance in glioma,^14^ nasopharyngeal carcinoma,^15^ and triple‐negative breast cancer.^16^ However, the role of ITGA5 in cervical cancer has been rarely reported thus far.

In present study, immunohistochemistry suggested that ITGA5 is closely associated with the OS and progression of cervical cancer patients. Furthermore, experimental models *in vitro* suggest that ITGA5 promotes angiogenesis in cervical cancer via the AKT/VEGFA pathway. These findings provide new insights that targeting ITGA5 may be a potential antiangiogenic strategy against cervical cancer to improve the prognosis of patients.

## 2 METHODS

### 2.1 Public RNA‐sequencing data analysis

In The Cancer Genome Atlas (TCGA) database, we searched the expression of 19 integrin α and β superfamily members of 306 cervical cancer patients and collected their clinical information. Then we calculated the hazard ratio of 19 integrin genes with OS in cervical cancer patients. Kaplan–Meier plotter (http://kmplot.com/analysis/) was performed to validate the correlation of *ITGA5* levels with OS in 304 cervical cancer patients based TCGA cohort. GSE168009 (including four concurrent chemoradiotherapy cervical cancer patients with no durable benefit and five patients with durable clinical benefit) and the corresponding platform annotation files were obtained from the Gene Expression Omnibus (GEO) database to analyze the expression of integrin superfamily members. Univariate and multivariate Cox regression analyses in 306 cervical cancer patients from TCGA database were performed by using the survival package in R environment. Prognostic nomogram was performed using the “rms” R package. A prognostic nomogram for predicting survival rates at 1‐, 2‐, and 3‐year of cervical cancer patients constructed based on *ITGA5* expression, tumor stage, and node stage from TCGA database. Total points were calculated by adding the points of the *ITGA5* expression and risk factors. Nomogram prediction performance of 1‐, 2‐, and 3‐year was conducted and calibration curves the more closer to the 45° reference line, nomogram‐predicted survival more closely corresponded with actual survival outcomes.

### 2.2 Gene Expression Profiling Interactive Analysis 2 (GEPIA2) database

GEPIA2 (http://gepia2.cancer‐pku.cn/) was conducted to validate the correlation of *ITGA5* levels with OS in 292 cervical cancer patients based on TCGA dataset. We also used GEPIA2 to examine the correlation between *ITGA5* and pro‐angiogenesis genes or anti‐angiogenesis genes or *FN1* levels in cervical cancer based on TCGA database.

### 2.3 Human tissue samples and clinical data

In total, 155 human cervical cancer surgical specimens were involved. Among them, 96 specimens were obtained from The First Affiliated Hospital, Shihezi University School of Medicine, and 59 were from Union Hospital, Tongji Medical College, Huazhong University of Science and Technology. All patients had pathologically confirmed diagnosis of primary cervical cancer and those undergoing prior chemotherapy or radiotherapy were excluded. The patients received surgery between 2008 and 2020, and their clinicopathological characteristics of these patients, including age, 2008 FIGO stage, lymph node metastasis, lymph vascular space invasion, histologic grade, tumor size, and overall survival (the period interval from surgery until a cancer‐related death or to the last follow‐up when no event occurred) were collected from medical records. All procedures related to the clinical samples were approved by the institute Ethics Committees and informed consent was obtained from all patients.

### 2.4 Immunohistochemistry (IHC)

The formalin‐fixed, paraffin‐embedded cervical cancer tissues were used for IHC. IHC was performed in the same way as previously described.^17^ The 5 μm tissue slices were incubated with rabbit anti‐ITGA5 antibody (1:200 dilution, Abcam, ab150361) or rabbit anti‐CD31 antibody (1:50 dilution, Abcam, ab28364) overnight at 4°C. The target proteins were visualized using fresh 3,3‐diaminobenzidine (DAB, ASPEN) solution. An appearance of brown under the microscope indicated positive staining. Next, the tissue sections were stained with hematoxylin (Solarbio) and then dehydrated. The IHC analysis results were determined by multiplying the score under a microscope at 200 × magnification according to the following criteria: staining intensity (none = 0, weak = 1, moderate = 2, strong = 3) and staining area (<5% = 0, <25% = 1, 25% ~ 50% = 2, 51% ~ 75% = 3, >75% = 4). Based on a median score of 6 in all tissues, we defined a score greater than 6 as high ITGA5 expression; otherwise, low ITGA5 expression was noted.

### 2.5 Cell culture, transfection, and processing

HeLa cells (cervical adenocarcinoma, CADC), SiHa cells (cervical squamous cell carcinoma, CSCC), and HUVECs (human umbilical vein endothelial cells) were purchased from American Type Culture Collection and cultured in RPMI‐1640 media containing 10% FBS (fetal bovine serum; GIBCO, America) at 37°C with 5% CO_2_. Short tandem repeat analysis was used to authenticate all cell lines within 2 years. *ITGA5* small interfering RNAs (siRNAs) were purchased from Tsingke Biotechnology Co. Ltd. and *FN1* siRNAs were purchased from RiboBio, all target sequences for the siRNAs are listed in Tables S1 and S2. Transfection was performed using Lipofectamine 2000 (Invitrogen) based on the manufacturer's protocol and cells transfected non‐targeting siRNA as negative control (NC).

To assess the influence of AKT pathway on the expression of VEGFA in cervical cancer cells, HeLa and SiHa cells were cultured with 10 and 15 μM MK‐2206 2HCl (Akt inhibitor, Selleck Chemicals) for 72 h for subsequent experiments.

To explore the role of fibronectin on the ITGA5‐mediated angiogenesis, before seeding the cervical cancer cells, non‐treated flat‐bottom plates were coated with 10 μg/mL of fibronectin (F2006, Sigma‐Aldrich) or not overnight at 4°C, respectively. After 3 days of culture, cells were used for corresponding experiments.

### 2.6 RNA extraction and quantitative real‐time PCR (qRT–PCR)

The total cellular RNA was extracted using TRIzol reagent (TaKaRa), followed by reverse transcription (HiScript®III RT SuperMix Vazyme). The primers were synthesized by Tsingke Biotechnology Co. Ltd., and their sequences are shown in Table S3. qRT–PCR assays were carried out with qPCR SYBR Green Master Mix (Vazyme) on a Step‐One Plus Real‐Time PCR System (ThermoFisher). Three biological replicates were performed.

### 2.7 Western Blotting

The total cellular protein extraction and Western Blotting were performed according to the previous description.^17^, ^18^ The antibodies used in Western blotting are listed in Table S4. The gray value of protein bands was examined by ImageJ (ImageJ software, National Institutes of Health, Bethesda, MD, USA). Each experiment was performed at least three times.

### 2.8 5‐Ethynyl‐20‐Deoxyuridine proliferation (EdU) assay

EdU assay was performed according to the manufacturer's instructions of the EdU experiment kit (RiboBio). Transfected cells were inoculated into a 96‐well plate at a density of 10,000 cells per well. The proportion of EdU‐positive cells was the proportion of proliferating cells. Images were acquired by fluorescence microscopy (Olympus). The results were obtained from three biological replicates. Each experiment was repeated in triplicate.

### 2.9 Cell counting kit‐8 (CCK‐8) assay

Transfected cells were seeded in 96‐well plates at a density of 5000 cells per well. Cells were incubated with 100 μL fresh medium with 10 μL CCK8 (HY‐K0301, MCE) for 4 h at 37°C. Cell viability was detected every 24 h for 3 days. Absorbance was measured at 450 nm using an ELx800 Absorbance Microplate Reader (BioTek). The experiment was performed in three biological replicates.

### 2.10 Apoptosis analysis

Apoptosis in cervical cancer cells was detected using the Annexin V‐PE/7‐AAD Apoptosis Detection Kit (BD Biosciences) according to the manufacturer's instructions. The cells were examined using a Sony ID7000 cell sorter (Sony). The number of early apoptotic cells, late apoptotic cells, or necrotic cells was determined by counting the percentage of Annexin V‐PE+/7‐AAD− cells (quadrant 3, Q3), Annexin V‐PE+/7‐AAD+ cells (Q2) or Annexin V‐PE−/7‐AAD+ cells (Q1), respectively (“+” indicates positive staining; “−” indicates negative staining). Annexin V‐PE−/7‐AAD− cells (Q4) were considered surviving cells.^19^ The percent of apoptosis cells is Q2 plus Q3. The experiment was performed in three biological replicates.

### 2.11 Identification of differentially expressed genes (DEGs) and functional enrichment analysis

Filtered by a *p* value <0.05 and fold change >1, DEGs between cervical cancer patients with high *ITGA5* level and low were screened using DESeq2 based on TCGA dataset (code CESC). Then, we acquired the hallmark gene sets from the Molecular Signatures Database and performed gene set enrichment analysis (GSEA) in DEGs. The correlation between genes and angiogenesis pathway scores was conducted by Spearman correlation through the R software GSVA package. Kyoto Encyclopedia of Genes and Genomes (KEGG) enrichment was analyzed using the clusterProfiler package.

### 2.12 Assessment of microvessel density (MVD)

Anti‐CD31 staining of cervical cancer sections was used to detect microvessel hot spots. Four selected hot spots in 200 × fields in areas with the most vascularized were counted for microvessel density, and any cluster of endothelial cells, even without lumens, was calculated.

### 2.13 Tube formation assay

To acquire conditioned medium (CM), cervical cancer cells with different treatment were flushed in PBS three times and cultured with serum‐free RPMI 1640 medium for 48 h. The supernatant was collected, and the cell fragments were removed through 5 min of centrifugation at 12,000 *g*. To further explore the role of VEGF in ITGA5‐mediated angiogenesis, we used VEGFR inhibitor bevacizumab (10 mg/mL, Roche Diagnostics GmbH) and 50 ng/mL recombinant human VEGFA protein (293‐VE, R&D Systems) to manipulate the VEGF signaling. They were added to the culture system of HUVECs containing CM from NC and si‐ITGA5 cervical cancer cells. HUVECs were precultured in CM for 48 h. A 96‐well plate was coated with 50 μL Matrigel (Corning) before HUVECs were seeded into wells (20,000 cells/well), and then 200 μL CM was added. Next, the cells were stained with calcein‐AM (Invitrogen) for 10 min after incubation for 6 h, and images were acquired by fluorescence microscopy (Olympus). ImageJ was used to quantify tube formation in this study. Each experiment was repeated in triplicate.

### 2.14 3D (three‐dimensional) spheroid sprouting assay

HUVECs were precultured in CM for 48 h. Spheroid sprouting assay was conducted following established protocols.^20^ In brief, a total of 3000 HUVECs were added to each well of a 96 U‐well suspension plate (Corning) in 100 μL of spheroid culture medium as described previously,^19^ with 20% Methocel (Solarbio). Cells formed spheroids for 48 h at 37°C. Then a solution of 3 mg/mL of rat tail collagen type I (Absin) was prepared in spheroid culture medium to 100 μL, spheroids were suspended with solution of collagen type I. After incubated at 37°C for 1.5 h to set the collagen gels, added 100 μL CM to each well and sprouts formed 48 h later. Images were taken on microscope and average sprout length was measured by ImageJ.

### 2.15 Single‐cell RNA‐seq analysis

Single‐cell transcriptome files of GSE168652 and GSE171894 were downloaded from GEO. For GSE168652, a single cell RNA sequencing on cervical tumor tissue and normal adjacent tissue from a cervical patient, we first detected the expression of *ITGA5*, *ANGPT1*, *ANGPT2*, and *VEGFA* in tumor cell subpopulation. Next, the correlation of *ITGA5* and *VEGFA* was conducted by Pearson correlation test. We also extracted the *ITGA5*‐posistive and *ITGA5*‐negative clusters in tumor cells and then identify DEGs to perform KEGG enriched pathway analysis. For GSE171894, a single‐cell RNA sequencing on tumor tissues from HPV16+ and HPV− cervical cancer patients, we extracted tumor cell cluster and calculated the expression proportion of different subpopulation tumor cell in HPV positive and negative tumor cells, respectively. Then we detected the expression of *ITGA5*, *ANGPT1*, *ANGPT2*, and *VEGFA* in different tumor cell clusters. A single‐cell RNA‐seq analysis was carried out with the Seurat package in R environment.

### 2.16 Enzyme‐linked immunosorbent assay (ELISA)

ELISA kits of human VEGFA (Elabscience) was purchased. Added 100 μL CM to each well and incubated for 90 min at 37°C. Next, Biotinylated VEGFA antibody (1:100) was added and incubated 1 h at 37°C. Then 100 μL HRP Conjugate working solution was added to wells and incubated for 30 min at 37°C. Added 90 μL Substrate Reagent to each well and incubated for 15 min at 37°C. Finally, added 50 μL Stop Solution to wells and immediately determine absorbance at 450 nm by ELx800 Absorbance Microplate Reader (BioTek).

### 2.17 Immunofluorescence

Cervical cancer cells were seeded in 24‐well plate at a density of 30,000 cells per well and incubated at 37°C overnight. Then plate was fixed with 4% paraformaldehyde for 30 min. After three times washed with PBS, 1% BSA for 2 h to block the antigen at room temperature and then incubated with primary antibody (FN1, 1:50 dilution, ABclonal, A12977) at 4°C overnight. Next, the plate was incubated with secondary antibody (Goat anti‐Rabbit IgG Cy3, 1:600 dilution, ab6939, Abcam) for 2 h at room temperature, followed by DAPI for 30 min to stain nuclei. Images were acquired by fluorescence microscopy (Olympus) and ImageJ was used to quantify the mean fluorescence intensity.

### 2.18 Statistical analysis

All statistical analyses were conducted using the R environment (version 4.1.0) and GraphPad Prism (version 9.0). The Kaplan–Meier method was used to plot survival curves and the difference in OS of cervical cancer patients stratified by ITGA5 IHC score was tested by the Log‐rank test. A Sankey diagram were built by the R software package ggalluvial. The expression of ITGA5 in IHC to clinical pathological parameters was analyzed by the Mann–Whitney *U* test. Student's *t* test was used to evaluate the statistical significance of differences in experimental model between two different groups and one‐way ANOVA was used to evaluate multiple groups. The correlation analysis was evaluated by Spearman correlation test or Pearson correlation test. A *p* value <0.05 was considered statistically significant.

## 3 RESULTS

### 3.1 ITGA5 is associated with a poor prognosis in cervical cancer patients

**FIGURE 1:**
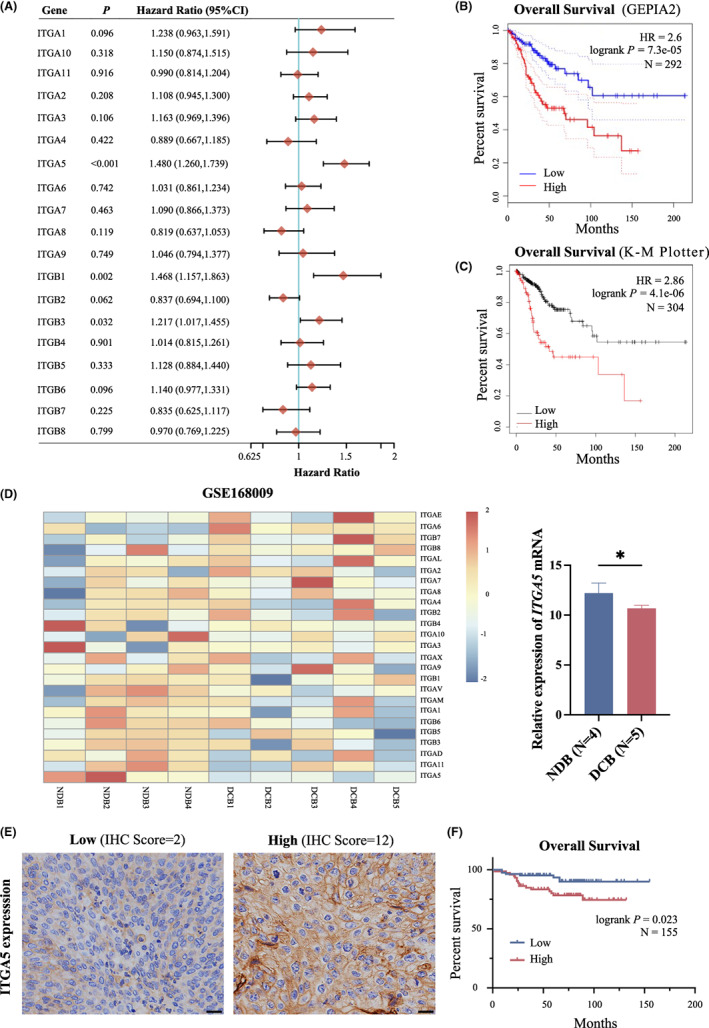
High *ITGA5* expression is associated with poorer prognosis in cervical cancer patients. (A) Forest plots of univariate Cox regression analyses involving integrin superfamily members in overall survival (OS) of 306 cervical cancer patients in TCGA dataset. (B) Kaplan–Meier curves of OS of cervical cancer patients with *ITGA5* high versus *ITGA5* low tumors using GEPIA2 data based on TCGA (*N* = 292). (C) Kaplan–Meier curves of OS of cervical cancer patients with *ITGA5* high versus *ITGA5* low tumors using Kaplan–Meier plotter data based on TCGA (*N* = 304). (D) The expression of integrins in cervical cancer patients with different response to concurrent chemoradiotherapy in GSE168009 (NDB, no durable benefit; DCB, durable clinical benefit). Bar, SD; *p < 0.05, Student's *t*‐test. (E) Representative images of immunohistochemistry (IHC) staining for ITGA5 in cervical cancer tissues. Scale bar, 50 μm. (F) Kaplan–Meier curves of OS in cervical cancer patients stratified by ITGA5 IHC score. (A score greater than 6 as high ITGA5 expression; otherwise, low ITGA5 expression was noted).

We first analyzed the prognostic significance of integrin α and β superfamily members in predicting the overall survival (OS) of cervical cancer patients in the TCGA dataset. We found that increased *ITGA5*, *ITGB1*, and *ITGB3* expression levels were significantly correlated with decreased OS, with *ITGA5* showing the highest risk for poorer OS (HR = 1.480 (1.260, 1.739); Figure 1A). Both GEPIA2 and Kaplan–Meier plotter analyses showed a significantly poorer OS in cervical cancer patients with high *ITGA5* expression (Figure 1B,C). According to the expression heatmaps of integrins in GSE168009 dataset, which includes four cervical cancer patients with no durable benefit from concurrent chemoradiotherapy and five patients with durable clinical benefit, *ITGA5* expression was significantly higher in the no benefit group (Figure 1D). Furthermore, based on TCGA cohort, univariate and multivariate Cox regression analyses showed that *ITGA5* level, tumor size, and lymph node metastasis were independent predictive factors for OS of cervical cancer patients (Figure S1A). We integrated these three factors into a prognostic nomogram using TCGA data to predict 1‐, 2‐, and 3‐year OS of cervical cancer patients; it exhibited a perfect prediction performance as revealed by the calibration curves (Figure S1B,C). Moreover, we performed IHC staining of ITGA5 in 155 cervical cancer tissues. The results showed expression heterogeneity with a staining score varying between 1 and 12, with a median of 6 (Figure 1E). The patients with a high ITGA5 IHC score (>6) had a significantly worse OS rate than those with a lower ITGA5 IHC score (Figure 1F). Thus, these results indicate that ITGA5 is associated with a poor prognosis in patients with cervical cancer.

### 3.2 ITGA5 promotes the progression of cervical cancer

**FIGURE 2:**
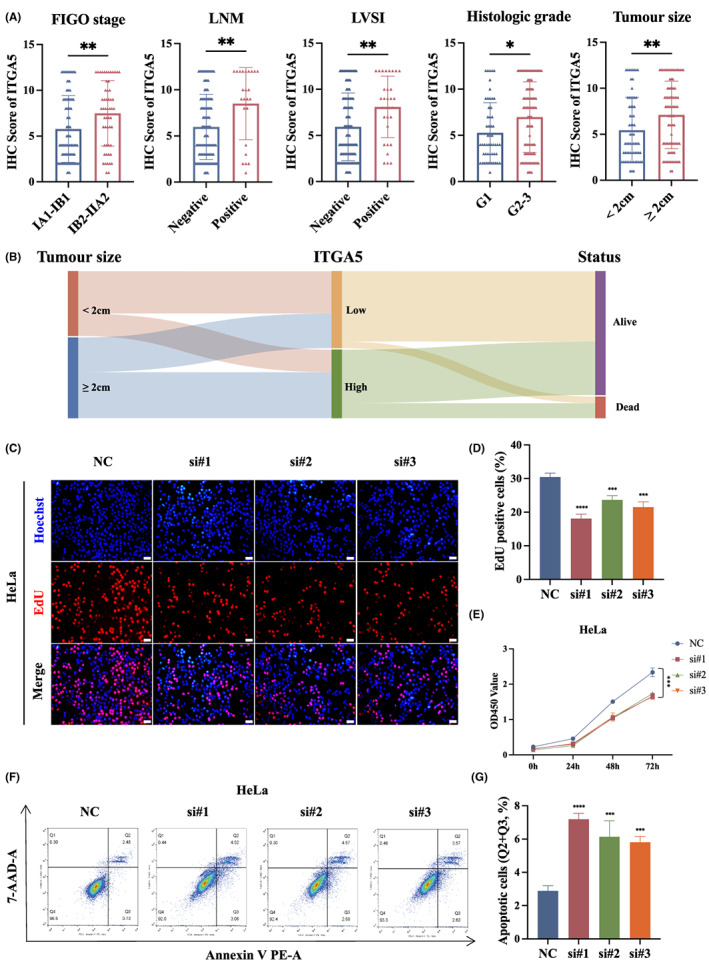
ITGA5 promotes progression of cervical cancer. (A) Scatter diagrams showing the distribution of ITGA5 IHC score in tumors grouped by FIGO stage, lymph node metastasis (LNM), lymph vascular space invasion (LVSI), histologic grade (G1, well differentiated tumor; G2‐3, moderate and poorly differentiated tumor), and tumor size. Bar, SD; Mann–Whitney *U* test. (B) The relationships among tumor size, level of ITGA5, and status of patients in the Sankey diagram. (C) Representative images of EdU assay of HeLa cells transfected with siITGA5 or negative control siRNA (NC). Scale bar, 50 μm. (D) The histogram shows the quantification results of the EdU assays of HeLa cells of three independent experiments. Bar, SD; One‐way ANOVA. (E) Cell proliferation curves generated with results of CCK‐8 assays of HeLa cells transfected with siITGA5 or NC of three independent experiments. One‐way ANOVA. (F) Representative images of flow cytometry apoptosis analysis of HeLa cells transfected with siITGA5 or NC. (G) The histogram shows the proportion of apoptotic HeLa cells (early apoptosis Q3 plus late apoptosis Q2) of three independent experiments. Bar, SD; One‐way ANOVA. *p < 0.05; **p < 0.01; ***p < 0.001; ****p < 0.0001.

To further explore the effects of ITGA5 in cervical cancer, we analyzed the correlation between ITGA5 IHC score and clinicopathological characteristics of patients. Our results suggested that ITGA5 expression was significantly associated with advanced FIGO stage (FIGO 2009, IB2‐IIA2), positive lymph node metastasis, positive lymph vascular space invasion, moderate or poor tumor differentiation, and large tumor size (≥2 cm in diameter) (Figure 2A; Table 1). Moreover, a Sankey diagram based on IHC score showed that larger tumor size was associated with high *ITGA5* and dead status of patients (Figure 2B). In addition, experiments *in vitro* were conducted to investigate the functional role of ITGA5 in cervical cancer. First, Western Blotting and qRT‐PCR assays were performed to evaluate interference efficiency of siRNAs on ITGA5 expression (Figure S2A). Next, as shown in EdU assays, the proliferative rates of HeLa cells were decreased after knockdown of ITGA5 (Figure 2C,D). Similarly, CCK‐8 assays showed significant inhibition of proliferation of HeLa cells transfected with siITGA5 compared to negative control (Figure 2E). Nevertheless, we did not observe this phenomenon in SiHa cells (Figure S2B,C). Annexin V binding assay showed that knockdown of ITGA5 increased the population of apoptotic cells in HeLa and SiHa cells (Figure 2F,G; Figure S2D). These findings suggest that ITGA5 participates in the progression of cervical cancer.

**TABLE 1:** Clinicopathological characteristics of patients with cervical cancer (*N* = 155).

Variables	*N*	ITGA5	
		High *N* (%)	Low *N* (%)
Age (years)			
Median (range)	47 (28–76)		
<50	94	45 (47.9)	49 (52.1)
≥50	61	28 (45.9)	33 (54.1)
FIGO stage			
IA1‐IB1	101	39 (38.6)	62 (61.4)
IB2‐IIA2	54	34 (63.0)	20 (37.0)
Lymph node metastasis			
Negative	131	55 (42.0)	76(58.0)
Positive	24	18 (75.0)	6 (25.0)
Lymph vascular space invasion			
Negative	124	50 (40.3)	74 (59.7)
Positive	31	23 (74.2)	8 (25.8)
Histologic grade			
G1	56	17 (30.4)	39 (69.6)
G2‐3	99	56 (56.6)	43 (43.4)
Tumor size			
<2 cm	69	24 (34.8)	45 (65.2)
≥2 cm	86	49 (57.0)	37 (43.0)

### 3.3 ITGA5 is correlated with angiogenesis in cervical cancer

**FIGURE 3:**
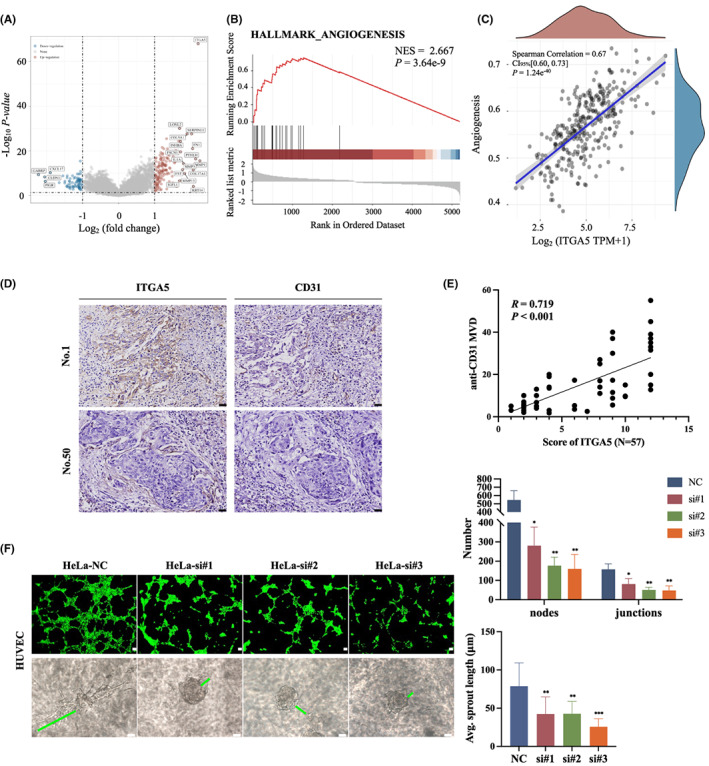
ITGA5 is correlated with angiogenesis in cervical cancer. (A) TCGA database volcano plot demonstrates differentially expressed genes (DEGs) between cervical cancer patients with different *ITGA5* expression (TCGA code CESC). (B) GSEA indicate that angiogenesis is significantly enriched in highly expressed *ITGA5* associated DEGs. (C) The correlations between *ITGA5* and angiogenesis pathway. Spearman correlation test. (D) Representative cases with high (NO.1) and low levels (NO.50) of ITGA5 that are correspondingly stained CD31 to identify microvessels are shown. Scale bar, 50 μm. (E) Correlation analysis of the immunohistochemistry score of ITGA5 and the microvessel density (MVD) in cervical cancer tissues (*N* = 57). Pearson correlation test. (F) Representative images of tube formation assays and 3D spheroid sprouting assays of HUVECs treated with conditional medium from HeLa cells transfected with siITGA5 or negative control siRNA (NC). The representative sprout is marked by green line. The histograms show the number of nodes and junctions of the tube formation assay and the average sprout length of 3D spheroid sprouting assay of three independent experiments. Scale bar, 50 μm. Bar, SD; One‐way ANOVA. *p < 0.05; **p < 0.01; ***p < 0.001.

In our study, RNA‐seq data were acquired from the TCGA database to identify differentially expressed genes (DEGs) between cervical cancer patients with high and low *ITGA5* expression levels, and a total of 217 significant DEGs, including 149 upregulated and 68 downregulated DEGs, were isolated (Figure 3A). GSEA indicated that angiogenesis was significantly enriched in the high *ITGA5* expression group (Figure 3B, Table S5). Moreover, *ITGA5* was significantly correlated with the global angiogenesis pathway signature (*p* < 0.001, *R* = 0.670; Figure 3C). As to individual genes involved in angiogenesis, *VEGFA*, *STC1*, *APP*, *ITGAV*, *VAV2*, *JAG2*, *OLR1*, *CCND2*, *POSTN*, *COL5A2*, *COL3A1*, *VCAN*, *FSTL1*, and *NRP1* were positively correlated with *ITGA5* (Fiure S3A). We also analyzed the correlation of *ITGA5* with anti‐angiogenesis genes *THBS2*, *PTEN*, *SERPZNF1*, and *IL‐10* in cervical cancer based on TCGA dataset (Figure S3B), but the correlations were rather modest as the *R* values ranged between −0.011 and 0.16, though the *p* values were less than 0.05. To confirm the correlation between ITGA5 and angiogenesis, we performed IHC for CD31 to identify microvessels in 57 human cervical cancers. We found that ITGA5 IHC score was positively correlated with the microvessel density MVD (*R* = 0.719, *p* < 0.001; Figure 3D,E). To test the function of ITGA5 in angiogenesis, we treated HUVECs with conditional medium (CM) from tumor cells transfected with siNC or siITGA5 and performed tube formation assays and 3D spheroid sprouting assays *in vitro*. The results revealed that ITGA5 knockdown in cervical cancer cells could significantly inhibit HUVECs to form tubes and sprouts (Figure 3F; Figure S3C). Overall, these results indicate a critical role of ITGA5 in cervical cancer angiogenesis.

### 3.4 ITGA5 promotes angiogenesis *in vitro* by regulating VEGFA

**FIGURE 4:**
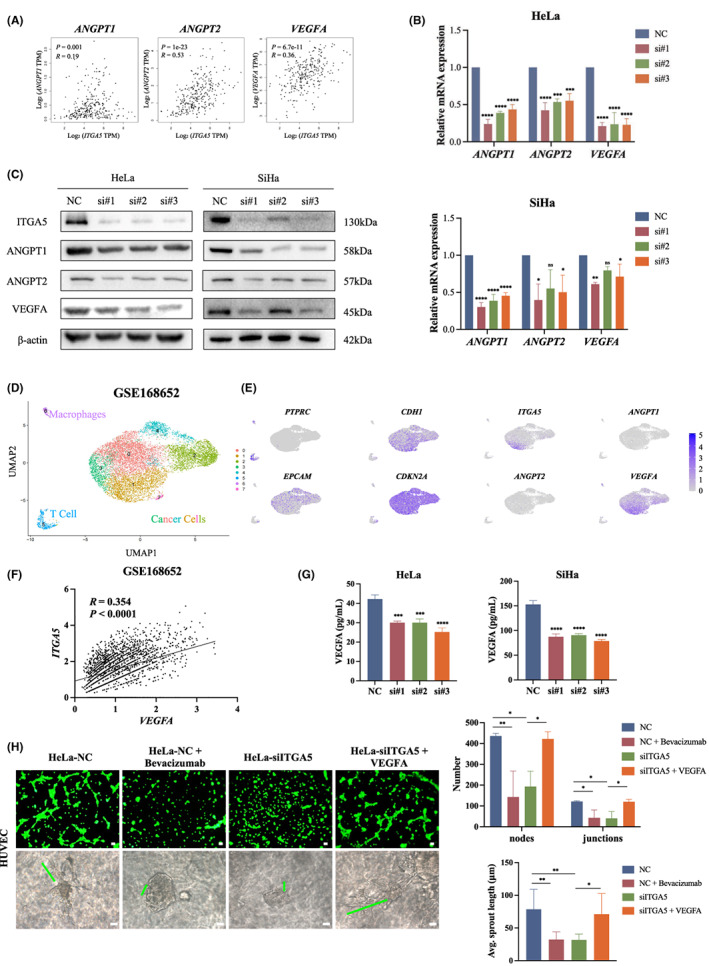
ITGA5 promotes angiogenesis in vitro by regulating VEGFA. (A) Correlation analysis between *ITGA5* expression and angiogenesis gene markers *ANGPT1*, *ANGPT2*, and *VEGFA* in cervical cancer by GEPIA2 based on TCGA dataset. Pearson correlation test. (B) qRT‐PCR for the mRNA level of *ANGPT1*, *ANGPT2*, *VEGFA* of HeLa and SiHa cells transfected with siITGA5 or negative control siRNA (NC). Three independent experiments. Bar, SD; One‐way ANOVA. (C) Western Blotting assay for the protein expression of ANGPT1, ANGPT2, VEGFA of HeLa and SiHa cells transfected with siITGA5 or NC. (D) Single‐cell RNA‐seq data of cell‐type and molecular subtype assignment of GSE168652, UMAP of cells from tumor tissue of cervical cancer patients, colored by clustering results. (E) Feature plots of relevant marker genes in immune cells (*PTPRC*) and cancer cells (*CDH1*, *CDKN2A*, and *EPCAM*) and the expression of *ITGA5*, *ANGPT1*, *ANGPT2*, and *VEGFA* in clusters of cancer cells. (F) Correlation analysis between *ITGA5* and *VEGFA* expression of cervical cancer cells in single‐cell RNA‐seq data of GSE168652. Pearson correlation test. (G) ELISA assay for the level of VEGFA in conditional medium from cervical cancer cells transfected with siITGA5 or NC of three independent experiments. Bar, SD; One‐way ANOVA. (H) Representative images of tube formation assay and 3D spheroid sprouting assay of HUVECs stimulated with NC conditional medium, NC conditional medium + Bevacizumab, siITGA5 conditional medium, and siITGA5 conditional medium + VEGFA in HeLa cells. The representative sprout is marked by green line. The histograms show the number of nodes and junctions of the tube formation assay and the average sprout length of 3D spheroid sprouting assay of three independent experiments. Scale bar, 50 μm. Bar, SD; One‐way ANOVA; *p < 0.05; **p < 0.01; ***p < 0.001; ****p < 0.0001; ns, not significant.

To further investigate the role of ITGA5 in the angiogenesis in cervical cancer, GEPIA2 based on TCGA dataset was used to explore the correlation between *ITGA5* and pro‐angiogenic genes *ANGPT1*, *ANGPT2*, and *VEGFA*. All these three genes were positively correlated with *ITGA5* (Figure 4A). In tumor cells with ITGA5 knockdown, qRT‐PCR and Western Blotting revealed decreased expression of *ANGPT1*, *ANGPT2*, and *VEGFA*, when compared to control cells. (Figure 4B,C; Figure S4A). Furthermore, based on the GSE168652 dataset of single‐cell RNA sequencing results of human cervical cancer tissues, we recognized six cancer cell subclusters, which were characterized by negative *PTPRC* (a marker of immune cells) and positive *CDH1*, *CDKN2A*, and *EPCAM* (markers of epithelial cells). *ITGA5* and *VEGFA* were coexpressed in clusters 1 and 3, while *ANGPT1* and *ANGPT2* were minimally expressed in all tumor cells (Figure 4D,E). The correlation of *ITGA5* and *VEGFA* expression in GSE168652 was significantly positive in a Pearson test (Figure 4F). Considering that VEGFA is an exocrine protein, we performed ELISA to examine the VEGFA in CM of HeLa and SiHa cells and found the levels of VEGFA were decreased after transfection with siITGA5 compared to control (Figure 4G). To investigate the role of VEGFA in the ITGA5‐induced angiogenesis, monoclonal antibody against VEGF Bevacizumab and exogenous VEGFA were used to manipulate the VEGF pathway in the culture system of HUVECs with cancer cell CM for tube formation assays and sprouting assays. In these assays, Bevacizumab could replicate the phenotype of ITGA5 knockdown, and exogenous VEFGA could largely reverse the ITGA5 knockdown‐induced inhibition of angiogenesis (Figure 4H; Figure S4B). Given that HPV has been associated with angiogenesis in cervical cancer,^21^ we analyzed the *ITGA5* expression in HPV‐positive versus HPV‐negative cervical cancers using GSE171894 single‐cell RNA‐seq dataset (Figure S5A–C). Among the five tumor cell clusters, we found that *ITGA5* and *VEGFA* were coexpressed in tumor clusters (C0‐C4), C0/C2 and C1/C3 were predominant in HPV‐positive and HPV‐negative tumors, respectively (Figure S5D). A coexpression of ITGA5 and VEGFA could be observed in C0, C2, and C3, while *ANGPT1* and *ANGPT2* expression was mainly found in C2 with extremely low abundance (Figure S5E), indicating a possible role of ITGA5/VEGFA axis in HPV‐induced angiogenesis. These findings suggest that ITGA5 promotes angiogenesis *in vitro* by regulating VEGFA in cervical cancer.

### 3.5 ITGA5 regulates the AKT/VEGFA signaling pathway

**FIGURE 5:**
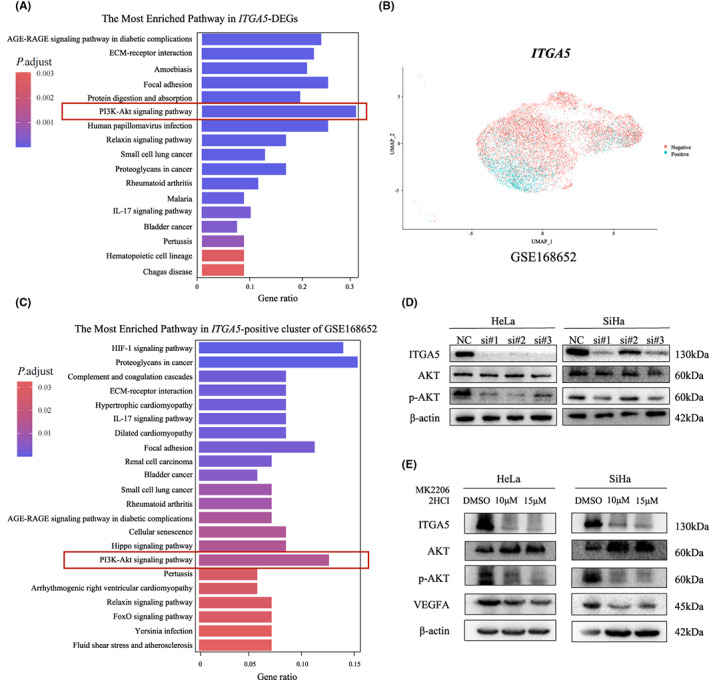
ITGA5 regulates the AKT/VEGFA signaling pathway. (A) KEGG enriched pathway analysis in ITGA5‐associated differentially expressed genes from TCGA. (B) UMAP of *ITGA5*‐positive and *ITGA5*‐negative cells from tumor tissue of cervical cancer patients in GSE168652, colored by clustering results. (C) KEGG enriched pathway analysis in *ITGA5*‐positive cluster of GSE168652. (D) Western Blotting assay for the expression of AKT and p‐AKT of HeLa and SiHa cells transfected with siITGA5 or negative control siRNA (NC). (E) Western Blotting assay show the expression ITGA5, AKT, p‐AKT, and VEGFA in HeLa and SiHa cells treated with DMSO, 10 μM, and 15 μM MK‐2206 2HCI (AKT inhibitor).

To elucidate the underlying mechanism of ITGA5 and angiogenesis in cervical cancer, we performed KEGG enriched pathway analysis of ITGA5‐associated DEGs identified in TCGA and the single cell‐sequencing data of *ITGA5*‐positive cells obtained from GSE168652, and the PI3K‐Akt signaling pathway was significantly enriched in both datasets (Figure 5A–C). However, the angiogenesis pathway is absent, which may be due to the disparities of algorisms used in KEGG and GSEA. Next, we assessed the AKT and phosphorylated AKT (p‐AKT) levels after ITGA5 knockdown using Western Blotting. We found that decreased ITGA5 expression inhibited the activation of p‐AKT in cervical cancer cells. (Figure 5D; Figure S6A). Moreover, we observed that the level of VEGFA in cervical cancer cells were significantly decreased after downregulation of p‐AKT by AKT inhibitor (MK2206 2HCI), confirming that the AKT pathway modulates VEGFA. Interestingly, the level of ITGA5 was also decreased in the cells treated with AKT inhibitor (Figure 5E; Figure S6B). These findings indicate that the AKT/VEGFA pathway might be the mechanism by which ITGA5 promotes cervical cancer angiogenesis.

### 3.6 Fibronectin plays a critical role in ITGA5‐mediated angiogenesis in vitro

**FIGURE 6:**
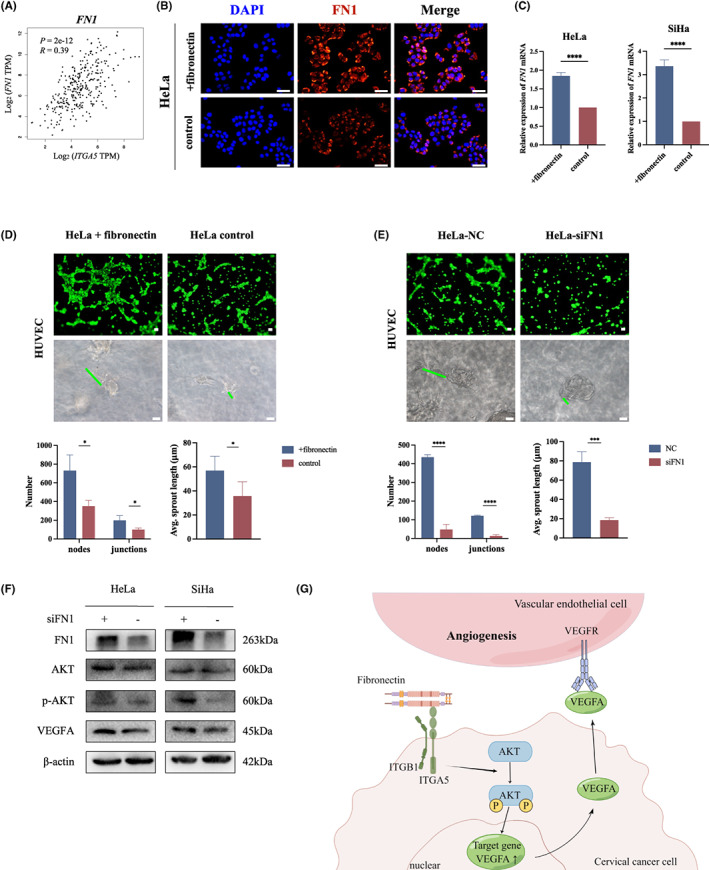
Fibronectin plays critical role in ITGA5‐mediated angiogenesis in vitro. (A) Correlation analysis between *ITGA5* expression and *FN1* in cervical cancer by GEPIA2 based on TCGA. Pearson correlation test. (B) Representative images of immunofluorescence staining of FN1 in HeLa cells with fibronectin substrate coated (+fibronectin) or control. Scale bar, 50 μm. (C) qRT‐PCR for the mRNA level of *FN1* of HeLa and SiHa cells coated with fibronectin substrate (+fibronectin) or control. Four independent experiments. Bar, SD; Student's *t*‐test. (D, E) Representative images of tube formation assay and 3D spheroid sprouting assay of HUVECs stimulated with conditional medium form HeLa cells with fibronectin substrate coated (+fibronectin) or control and conditional medium from HeLa cells transfected with siFN1 or negative control siRNA (NC), respectively. Scale bar, 50 μm. The representative sprout is marked by green line. The histograms show the number of nodes and junctions of the tube formation assay and the average sprout length of 3D spheroid sprouting assay of three independent experiments. Bar, SD; Student's *t*‐test. (F) Western Blotting assay show the expression of FN1, AKT, p‐AKT, and VEGFA in HeLa and SiHa cells transfected with siFN1 or NC. (G) The schematic figure shows that ITGA5 promotes angiogenesis in cervical cancer by AKT/VEGFA axis and Fibronectin plays critical role in this pathway. *p < 0.05; ***p < 0.001; ****p < 0.0001.

Since fibronectin (FN1) is the main ligand for ITGA5, we investigated the influence of the presence of FN1 on ITGA5‐mediated angiogenesis in cervical cancer. The results of pairwise gene correlation analysis of GEPIA2 data showed that the expression of *FN1* was positively related to *ITGA5* expression in cervical cancer (Figure 6A). We detected the FN1 expression in cervical cancer cells and cells cultured with FN1 substrate by immunofluorescence staining and qRT‐PCR, which showed that cervical cancer cells can secrete FN1 by themselves and a significant upregulation of FN1 expression in FN1 coated cells (Figure 6B,C; Figure S7A,B). To verify whether FN1 could promote ITGA5‐mediated angiogenesis *in vitro*, we downregulated *FN1* in cervical cancer cells transfected with siRNAs (siFN1 vs. siNC) or cultured cells in FN1‐containing substrate (+fibronectin vs. control) to manipulate the FN1‐ITGA5 interaction. CM samples from the cancer cells with downregulated or upregulated FN1 were used to treat HUVECs to perform tube formation assays and 3D spheroid sprouting assays. The results indicated that FN1 significantly enhanced the angiogenesis of HUVECs *in vitro* (Figure 6D,E; Figure S7C,D). Moreover, to further verify the influence of FN1 on AKT pathway and the expression of VEGFA in cervical cancer cells, we performed Western Blotting to evaluate the expression of AKT, p‐AKT, and VEGFA in the tumor cells transfected with siFN1 or siNC. We found that decreased FN1 expression inhibited the phosphorylation of AKT and the expression of VEGFA in cervical cancer cells (Figure 6F; Figure S7E). These results illustrate that fibronectin may play a critical role in ITGA5‐mediated angiogenesis.

## 4 DISCUSSION

Angiogenesis is an essential occurrence during tumor growth since they carry oxygen and nutrients to cells and thus are critical to cervical cancer development.^22^, ^23^ Anti‐angiogenic therapy is a promising strategy for treatment of inoperable cervical cancer. However, in advanced cervical cancer patients, the clinical response and benefits of VEGFR‐targeting therapy are limited.^24^ Thus, it is meaningful to explore further mechanism underlying angiogenesis, which may fuel new ideas for targeted therapy. Here, we identify ITGA5 as the most survival‐related integrin superfamily member in cervical cancer and unravel its role in sprouting angiogenesis and VEGFA expression regulation, suggesting that ITGA5 is a potential mediator of angiogenesis in cervical cancer.

In this study, clinical data analysis established that high expression of ITGA5 is significantly correlated with poorer OS in patients with cervical cancer, IHC analysis established that high ITGA5 level was associated with cervical cancer progression, instead of a bystander, which reveal that more intensive postoperative treatment, such as chemoradiotherapy or individualized treatment, is necessary for patients with high ITGA5 expression. Moreover, several preclinical studies suggested that integrins antagonists might be useful to suppress tumor angiogenesis and growth either alone or in combination with existing cancer therapeutics.^25^

Angiogenesis is an intricate process regulated by a series of molecules. The critical role of VEGFA in tumor angiogenesis has been widely studied.^26^ Our findings suggested that the angiogenic function of ITGA5 in cervical cancer was through increasing VEGFA expression. Additionally, our study revealed that AKT signaling participated in the ITGA5‐mediated regulation of VEGFA expression. The AKT signaling pathway as a key mechanism can affect angiogenesis through various ways to promote tumor progression, which have been demonstrated by previous reports.^27^, ^28^ Our results showed that the AKT pathway modulated VEGFA in cervical cancer cells. Similar findings have been reported in colorectal cancer.^29^ Besides, we observed that AKT inhibitor could significantly suppressed the expression of ITGA5 in cervical cancer cells, consistent with previously report that the AKT pathway had an obvious inhibitory effect on ITGA5 level in chorioretinal endothelial cells,^30^ indicating a positive feedback loop between ITGA5 and AKT‐signals, as ITGA5 is both upstream and downstream to AKT‐pathway.

Integrin is a cell adhesion receptor, and engagement with ECM ligands triggers the downstream signaling pathways, such as activation of the AKT pathway.^31^ AKT is often activated in response to environmental stresses influenced by the organization of the ECM.^32^ Integrins are heterodimeric receptors that comprise an α and a β‐subunit and provide attachment to the ECM and control responses to mechanical stimuli. The binding of ECM proteins to fibronectin (FN1) facilitates the maturation and tissue specificity of the ECM.^33^ As previous reports, fibronectin specific integrin–integrin α_5_β_1_ play complex roles in malignancy.^8^, ^34^ Given that, we investigated the influence of the presence of FN1 to ITGA5‐mediated angiogenesis in our study. We observed that FN1 overexpression promoted ITGA5‐mediated angiogenesis and downregulated FN1 in cervical cancer cells decreased angiogenesis by inhibited AKT signaling pathway *in vitro*, suggesting that fibronectin may play a critical role for ITGA5‐mediated angiogenesis *in vitro* by binding ITGA5 to regulate ECM features to activate AKT signaling pathway. However, fibronectin mediates a wide variety of cellular interactions with ECM, the function of pro‐angiogenic by FN1 overexpression may influence by various mechanisms and further studies are necessary to confirm our findings concerning the role of fibronectin on exact function of ITGA5. Taken together, our findings suggest a new molecular mechanism by which ITGA5, as an upstream mediator of VEGFA, promotes angiogenesis by regulating the AKT pathway, and fibronectin plays critical role in this function (Figure 6G).

However, the above conclusions have not been well substantiated, and to further confirm our findings, additional clinical samples need to be included. Moreover, future *in vivo* investigation of ITGA5‐mediated angiogenesis in cervical cancer is necessary and investigations are still required to identify the underlying mechanism.

## 5 CONCLUSIONS

In conclusion, our study reveals that ITGA5 promotes angiogenesis in cervical cancer and is associated with a poor prognosis in patients. The ITGA5/AKT/VEGFA axis may be a potential target for developing therapeutic approaches for angiogenesis and fibronectin may play critical role for this pathway. We propose that ITGA5 could serve as a novel promising molecular therapeutic target for treating patients with advanced cervical cancer.

## AUTHOR CONTRIBUTIONS

**Xiaohan Xu:** Conceptualization (supporting); data curation (equal); formal analysis (equal); investigation (equal); methodology (equal); resources (equal); software (equal); validation (equal); visualization (equal); writing – original draft (equal); writing – review and editing (equal). **Lulu Shen:** Data curation (equal); formal analysis (equal); investigation (equal); methodology (equal); resources (equal); software (equal); validation (equal); visualization (equal); writing – original draft (supporting). **Wenhan Li:** Data curation (equal); formal analysis (equal); investigation (equal); methodology (equal); resources (equal); software (lead); validation (equal); visualization (equal). **Xiaoli Liu:** Data curation (equal); formal analysis (equal); investigation (equal); methodology (equal); resources (equal); software (equal); validation (equal); visualization (equal); writing – review and editing (equal). **Ping Yang:** Conceptualization (equal); data curation (equal); funding acquisition (lead); project administration (equal); resources (equal); supervision (equal); validation (equal); visualization (equal); writing – review and editing (equal). **Jing Cai:** Conceptualization (lead); methodology (lead); project administration (lead); resources (equal); supervision (lead); validation (equal); visualization (equal); writing – original draft (equal); writing – review and editing (equal).

## FUNDING INFORMATION

Funding for this project has been provided by the Natural Science Foundation of China (Nos. 82072893).

## CONFLICT OF INTEREST STATEMENT

The authors declare that there is no conflict of interest.

## ETHICS APPROVAL STATEMENT

All procedures related to the clinical samples were approved by the Ethics Committee of The First Affiliated Hospital, Shihezi University School of Medicine and Ethics Committee of Tongji Medical College, Huazhong University of Science and Technology (KJX‐2021‐104‐01).

## Supporting information


Figure S1.
Figure S2.
Figure S3.
Figure S4.
Figure S5.
Figure S6.
Figure S7.

Table S1.
Table S2.
Table S3.
Table S4.

Table S5.

## Data Availability

The article includes all data created and/or analyzed during this work.
