# Supplementary material for: ITGA5 promotes tumor angiogenesis in cervical cancer
Source: Cancer Med. 2023 Mar 31;12(10):11983–99. doi: 10.1002/cam4.5873 (PMC10242342; doi:10.1002/cam4.5873)
Supplement: Supplementary file 1 — Figure S1. Figure S2. Figure S3. Figure S4. Figure S5. Figure S6. Figure S7. [file CAM4-12-11983-s003.pdf]

Supplementary Figure 1

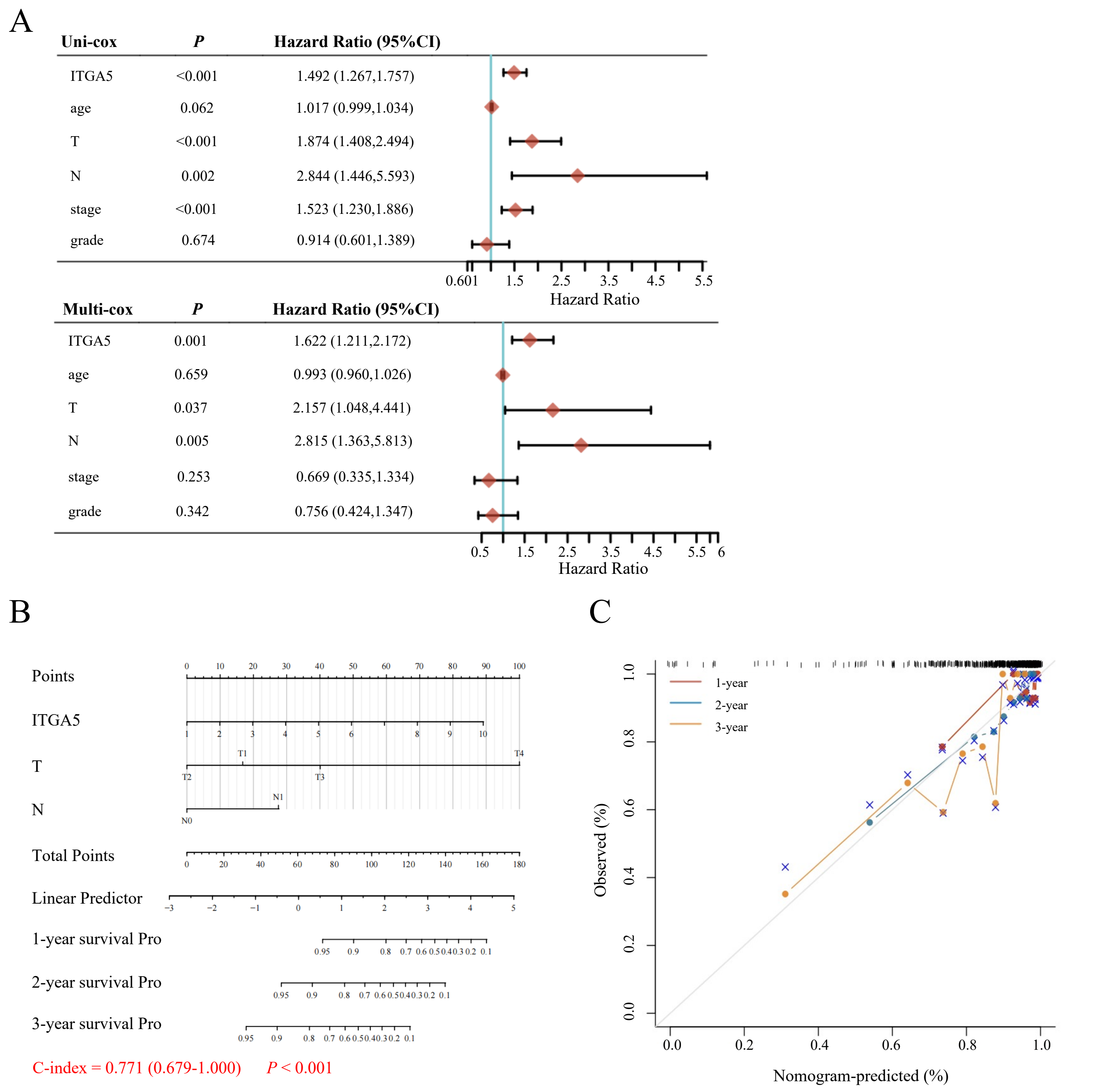

Supplementary Figure 1 The expression of *ITGA5* is associated with poorer prognosis of cervical cancer patients. (A) Forest plots of univariate and multivariate Cox regression analyses involving *ITGA5* level and clinical risk factors in 306 cervical cancer patients of TCGA database. **(B)** A Nomogram is constructed to predict the 1-, 2-, and 3-year of overall survival for patients with cervical cancer based on TCGA database. Total points were calculated by adding the points of the *ITGA5* expression and risk factors. **(C)** The calibration curves of 1-, 2-,and 3- year of prognostic prediction nomogram to verify the prediction accuracy. The curves more closer to the 45° reference line, the nomogram-predicted survival more closely corresponded with actual survival outcomes.

Supplementary Figure 2

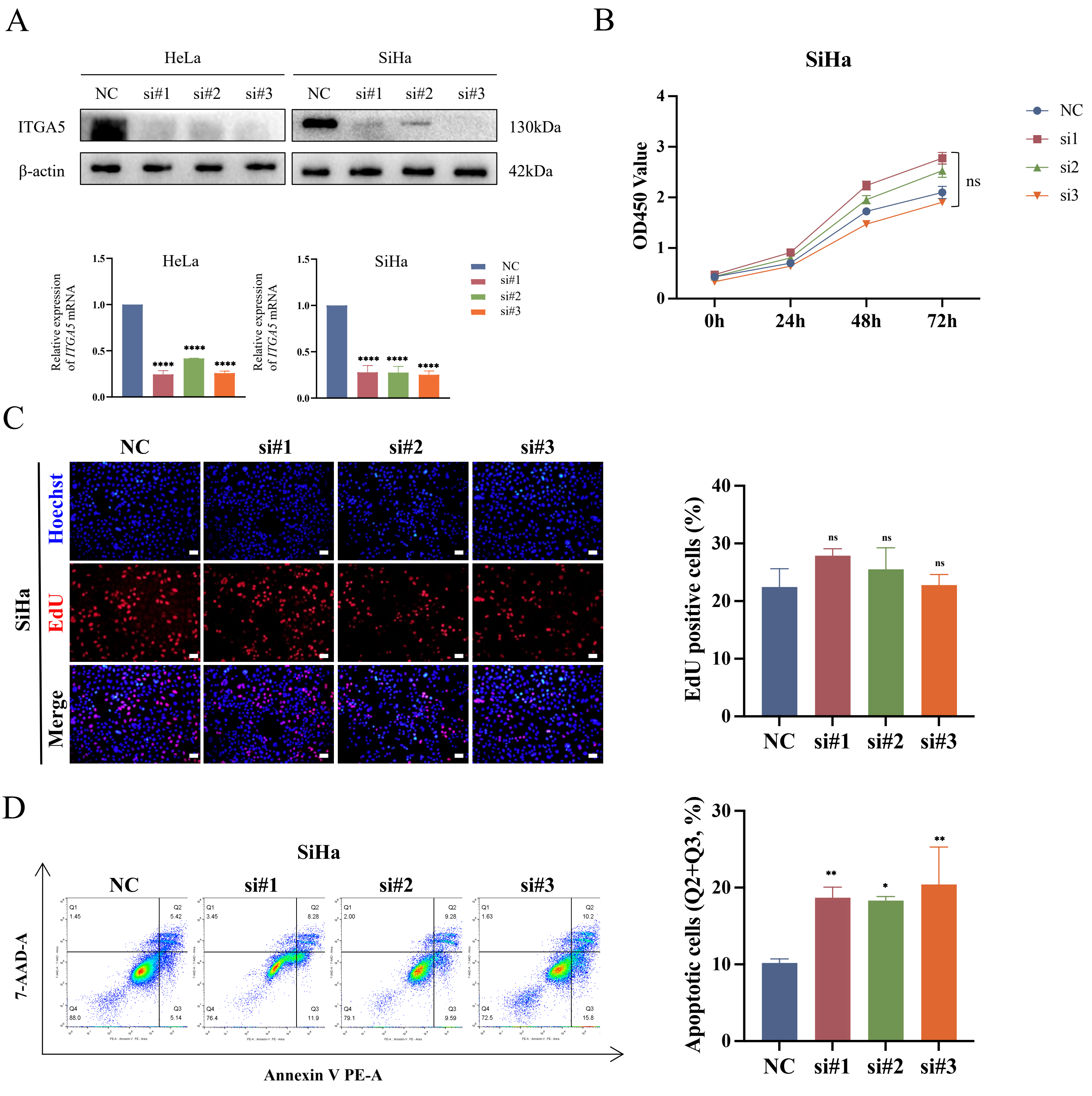

Supplementary Figure 2 ITGA5 promotes progression of cervical cancer. (A) Western Blotting and qRT-PCR indicated the siRNAs interference efficiency of ITGA5 in HeLa and SiHa cells. Three independent experiments. Bar, SD; One-way ANOVA. **(B)** Cell proliferation curves generated with results of CCK-8 assays of SiHa cells transfected with siITGA5 or negative control siRNA (NC) of three independent experiments. One-way ANOVA. **(C)** EdU assay of SiHa cells transfected with siITGA5 or NC. Scale bar, 50 μm. The histogram shows the quantification results of the EdU assays of SiHa cells of three independent experiments. Bar, SD; One-way ANOVA. **(D)** Representative images of flow cytometry apoptosis analysis of SiHa cells transfected with siITGA5 or NC. The histogram shows the proportion of apoptotic SiHa cells (early apoptosis Q3 plus late apoptosis Q2) of three independent experiments. Bar, SD; One-way ANOVA. *, $P < 0.05$; **, $P < 0.01$; ****, $P < 0.0001$; ns, not significant.

Supplementary Figure 3

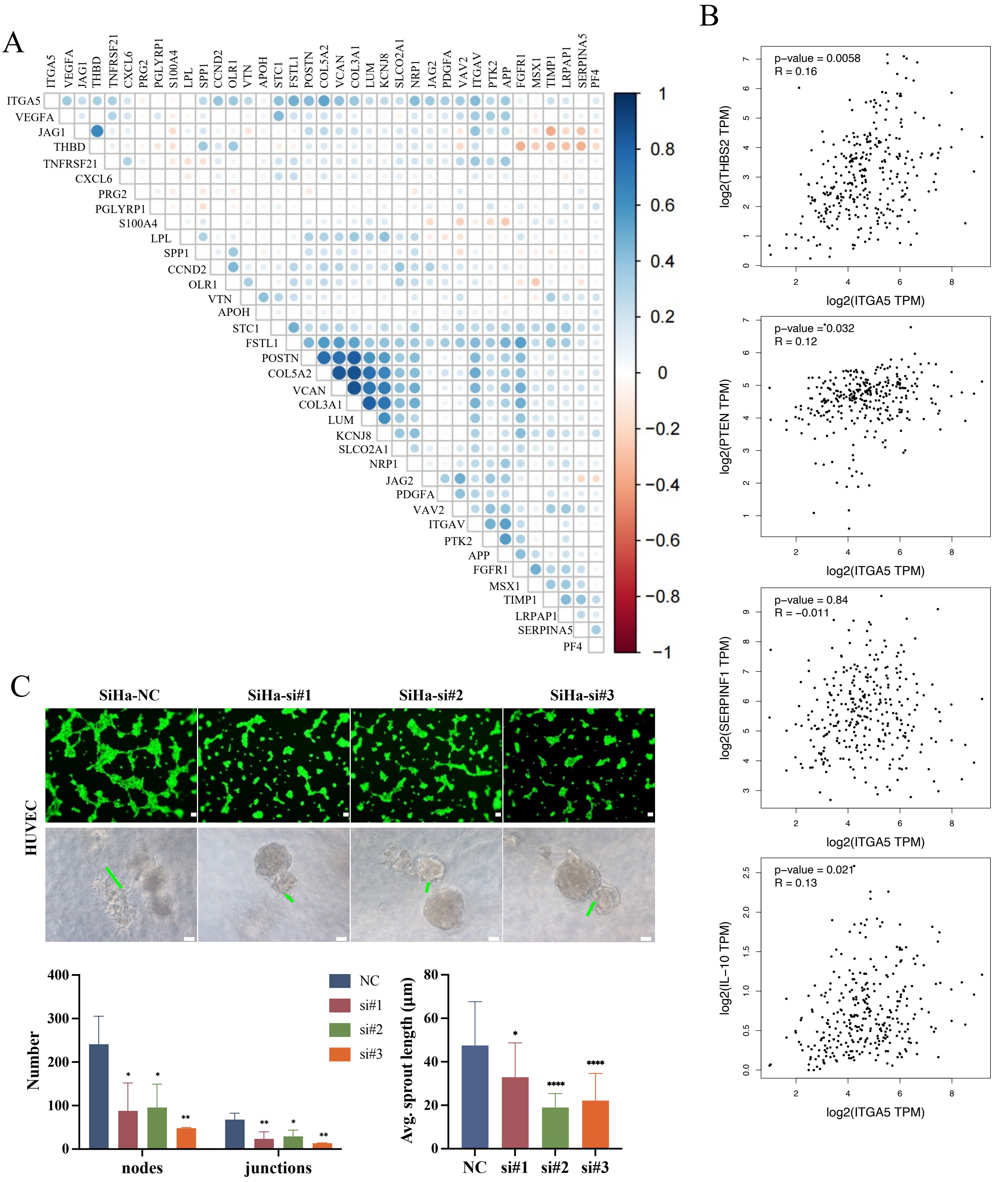

Supplementary Figure 3 ITGA5 is correlated with angiogenesis in cervical cancer. (A) The correlation of *ITGA5* and genes in angiogenesis pathway based on TCGA dataset. Spearman correlation test. **(B)** The correlation of *ITGA5* and anti-angiogenesis genes including *THBS2*, *PTEN*, *SERPZNF1*, and *IL-10* by GEPIA2 based on TCGA dataset. Pearson correlation test. **(C)** Representative images of tube formation assay and 3D spheroid sprouting assay of HUVECs stimulated with conditional medium from SiHa cells transfected with siITGA5 or negative control siRNA (NC). The representative sprout is marked by green line. The histograms show the number of nodes and junctions of the tube formation assay and the average sprout length of 3D spheroid sprouting assay of three independent experiments. Scale bar, 50 μm. Bar, SD; One-way ANOVA. *, $P < 0.05$; **, $P < 0.01$; ****, $P < 0.0001$.

Supplementary Figure 4

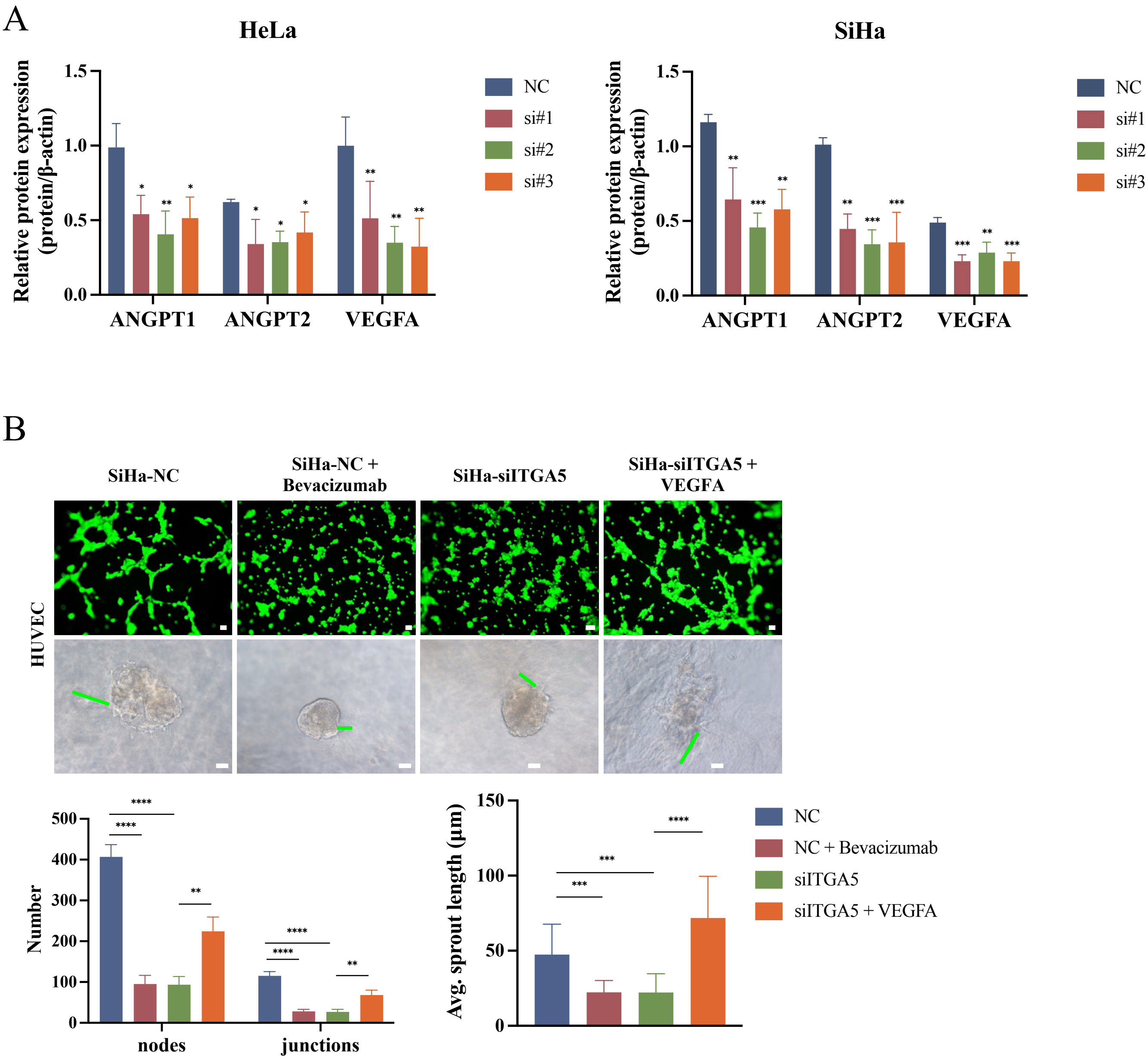

Supplementary Figure 4 ITGA5 promotes angiogenesis *in vitro* by regulating VEGFA. (A) The histograms show the gray value of ANGPT1, ANGPT2, and VEGFA in HeLa and SiHa cells transfected with siITGA5 or negative control siRNA (NC) of three independent experiments by Western Blotting. Bar, SD; One-way ANOVA. **(B)** Representative images of tube formation assay and 3D spheroid sprouting assay of HUVECs stimulated with NC conditional medium, NC conditional medium + Bevacizumab, siITGA5 conditional medium, and siITGA5 conditional medium + VEGFA in SiHa cells. The representative sprout is marked by green line. The histograms show the number of nodes and junctions of the tube formation assay and the average sprout length of 3D spheroid sprouting assay of three independent experiments. Scale bar, 50 μm. Bar, SD; One-way ANOVA. *, $P< 0.05$; **, $P< 0.01$; ***, $P< 0.001$; ****, $P< 0.0001$.

Supplementary Figure 5

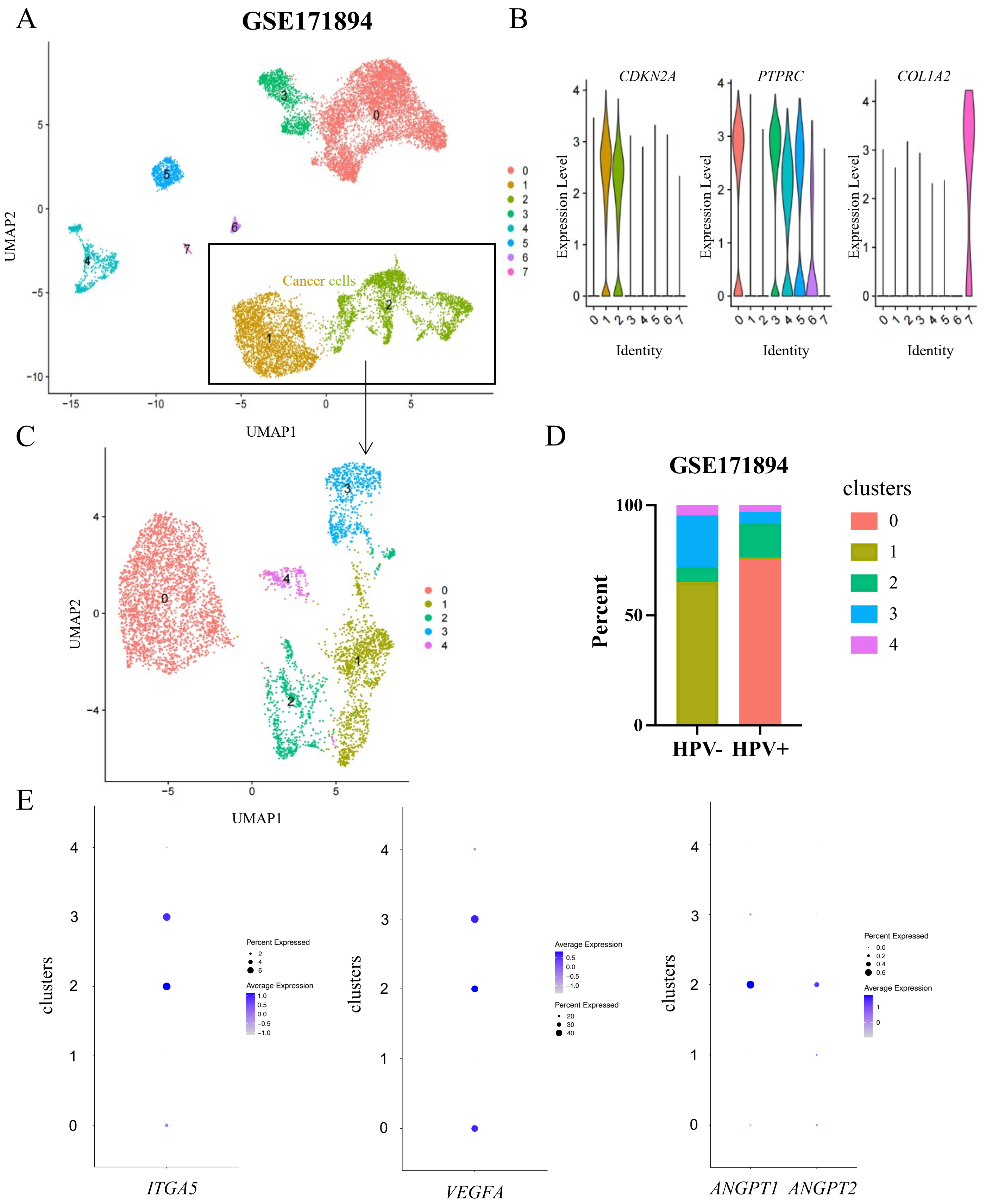

Supplementary Figure 5 Single-cell RNA-seq data of cell-type and molecular subtype assignment of GSE171894. (A-B) UMAP of cells from tumor tissue of HPV-positive and HPV-negative cervical cancer patients, colored by clustering results. Feature plots of relevant marker genes, *CDKN2A* in tumour cells, *PTPRC* in immune cells, and *COL1A2* in cancer-associated fibroblast, respectively. **(C)** UMAP of tumour cells extracted from cluster 1 and 2 in **A**, colored by clustering results. **(D)** The proportion of different subpopulation tumour cells in HPV-positive and HPV-negative cancer cells., colored by clusters. **(E)** The expression of *ITGA5*, *VEGFA*, *ANGPT1* and *ANGPT2* in subpopulation of tumour cells.

Supplementary Figure 6

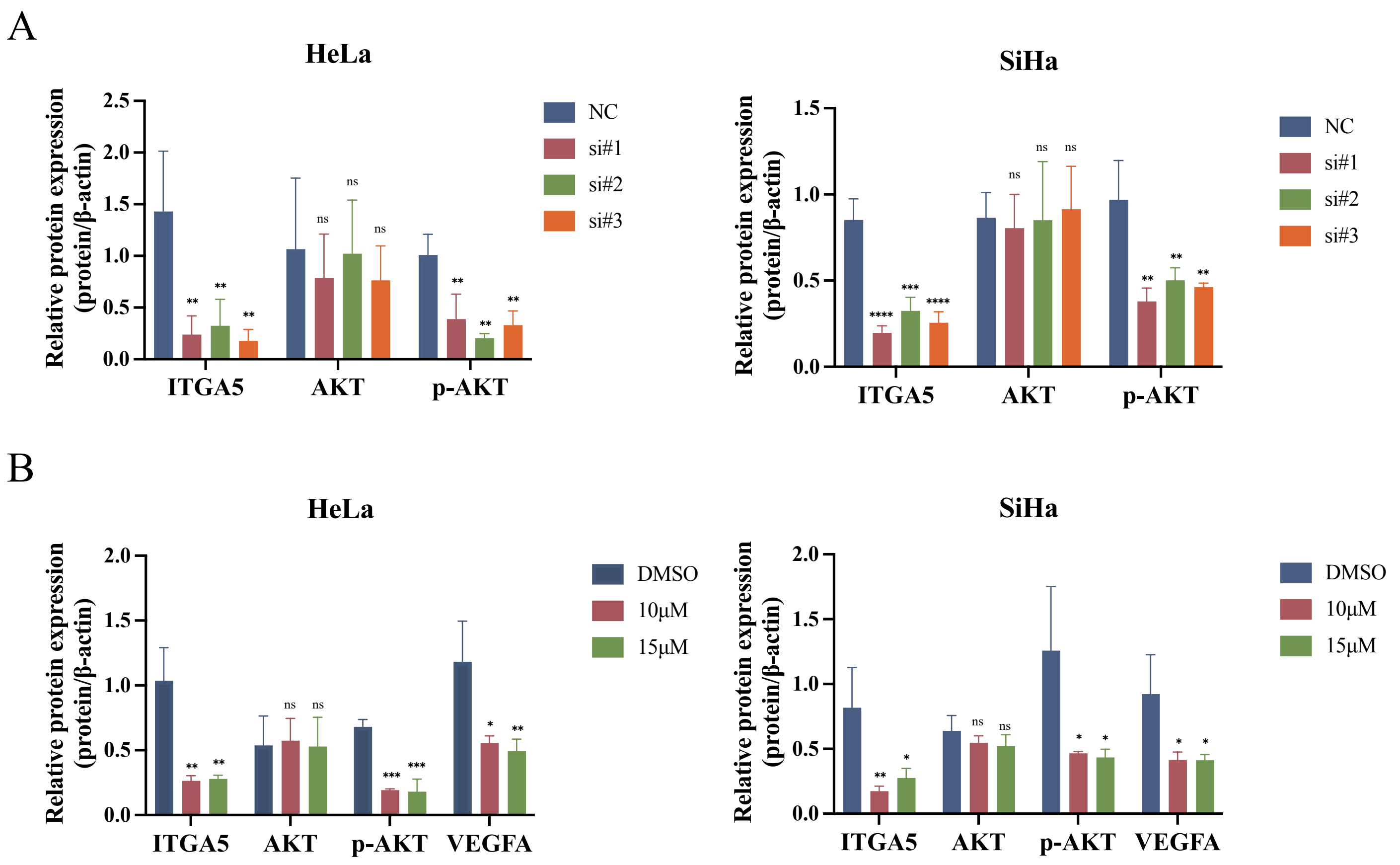

Supplementary Figure 6 ITGA5 regulates the AKT/VEGFA signaling pathway. (A) The histograms show the gray value of ITGA5, AKT, and p-AKT in HeLa and SiHa cells transfected with siITGA5 or negative control siRNA (NC) of three independent experiments by Western Blotting. Bar, SD; One-way ANOVA. **(B)** The histograms show the gray value of ITGA5, AKT, p-AKT, and VEGFA in HeLa and SiHa cells treated with DMSO control, 10μM, and 15μM MK-2206 2HCI (AKT inhibitor) of three independent experiments by Western Blotting. Bar, SD; One-way ANOVA. *, P< 0.05; **, P< 0.01; ***, P< 0.001; ****, P< 0.0001; ns, not significant.

Supplementary Figure 7

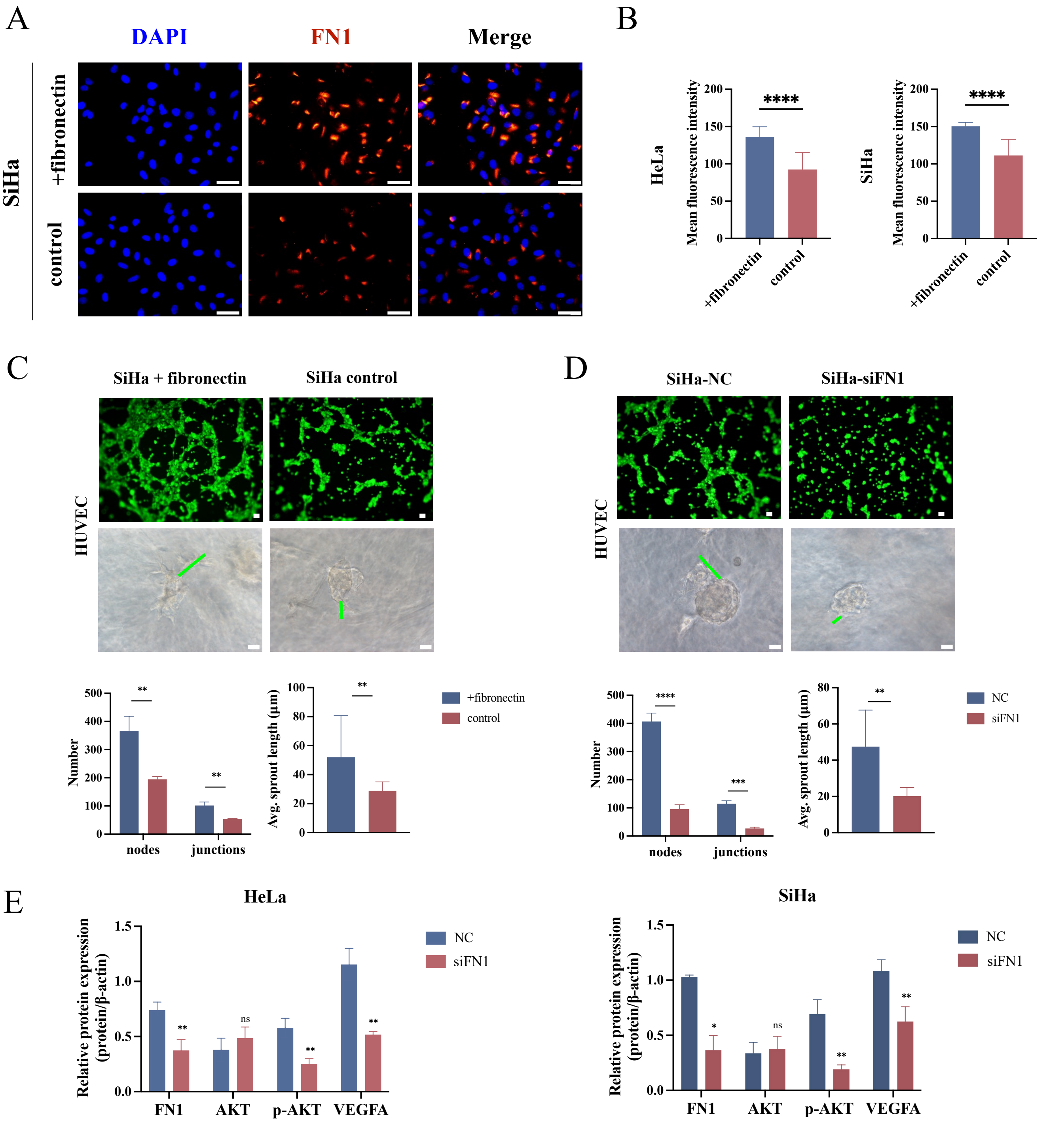

Supplementary Figure 7 Fibronectin plays critical role in ITGA5-mediated angiogenesis *in vitro*. (A) Representative images of immunofluorescence staining of fibronectin (FN1) in SiHa cells with fibronectin substrate coated (+fibronectin) and control. Scale bar, 50 μm. **(B)** The histograms show the mean fluorescence intensity of FN1 in HeLa and SiHa cells with fibronectin substrate coated (+fibronectin) or control of three independent experiments. Bar, SD; Student's t-test. **(C-D)** Representative images of tube formation assay and 3D spheroid sprouting assay of HUVECs stimulated with conditional medium from fibronectin substrate coated (+fibronectin) or control SiHa cells and conditional medium from SiHa cells transfected with siFN1 or negative control siRNA (NC), respectively. The representative sprout is marked by green line. The histograms show the number of nodes and junctions of the tube formation assay and the average sprout length of 3D spheroid sprouting assay of three independent experiments. Scale bar, 50 μm. Bar, SD; Student's t-test. **(E)** The histograms show the gray value of FN1, AKT, p-AKT, and VEGFA in HeLa and SiHa cells transfected with siFN1 or NC of three independent experiments by Western Blotting. Bar, SD; Student's t-test. *, $P< 0.05$; **, $P< 0.01$; ***, $P< 0.001$; ****, $P< 0.0001$; ns, not significant.
