# Supplementary material for: ITGA5 promotes tumor angiogenesis in cervical cancer
Source: Cancer Med. 2023 Mar 31;12(10):11983–99. doi: 10.1002/cam4.5873 (PMC10242342; doi:10.1002/cam4.5873)
Supplement: Supplementary file 2 — Table S1. Table S2. Table S3. Table S4. [file CAM4-12-11983-s002.doc]

**Supplementary Table 1 Sequence of siRNA for *ITGA5***

| **siRNA** | **Sequences** |
| --- | --- |
| **siRNA-1#** | GGACCAGGAAGCUAUUUCUTT |
| **siRNA-2#** | GCAGGGAGUAGUGUUUGUATT |
| **siRNA-3#** | CACCCGAAUUCUGGAGUAUTT |

**Supplementary Table 2 Sequence of siRNA for *FN1***

| **siRNA** | **Sequences** |
| --- | --- |
| **siRNA-FN1** | GCCAACCTTTACAGACCTA |

**Supplementary Table 3 Sequences of primer used for qRT-PCR**

| **Gene** | **Forward primer sequences** | **Reverse primer sequences** |
| --- | --- | --- |
| ***ITGA5*** | TTACGGGACTCAACTGCACC | AGCCTGAAACACTCAGCCTC |
| ***ANGPT1*** | GAAGGATGCTGATAACGACAAC | AGTTTTCCATGATTTTGTCCCG |
| ***ANGPT2*** | TGTATGATCACTTCTACCTCGC | TGGCTGATGCTACTTATTTTGC |
| ***VEGFA*** | AGGGCAGAATCATCACGAAGT | AGGGTCTCGATTGGATGGCA |
| ***FN1*** | AGGAAGCCGAGGTTTTAACTG | AGGACGCTCATAAGTGTCACC |
| ***β-actin*** | TACATGGCTGGGGTGTTGAA | AAGAGAGGCATCCTCACCCT |

**Supplementary Table 4 Antibodies used in western blot**

| **Antibodies** | **Cat No.** | **Dilution** |
| --- | --- | --- |
| **ITGA5** | Abcam, ab150361 | 1:500 |
| **ANGPT1** | ABclonal, A3757 | 1:1000 |
| **ANGPT2** | ABclonal, A11306 | 1:1000 |
| **VEGFA** | Proteintech, 19003-1-AP | 1:1000 |
| **AKT** | Cell Signaling Technology, 2920 | 1:2000 |
| **p-AKT** | Cell Signaling Technology, 4060 | 1:2000 |
| **FN1** | ABclonal, A12977 | 1:5000 |
| **β-actin** | Proteintech, 20536-1-AP | 1:1000 |
