# Supplementary material for: ITGA5 promotes tumor angiogenesis in cervical cancer
Source: Cancer Med. 2023 Mar 31;12(10):11983–99. doi: 10.1002/cam4.5873 (PMC10242342; doi:10.1002/cam4.5873)
Supplement: Supplementary file 3 — Table S5. [file CAM4-12-11983-s001.pdf]

Supplementary Table 5 GSEA hallmark of *ITGA5* differentially expressed genes

ID	setSize	enrichmentScore	NES	pvalue	p.adjust	qvalues	rank	leading_edge
HALLMARK_APICAL_JUNCTION	103	0.544319037	2.63820175	1.00E-10	7.00E-10	2.86E-10	1140	tags=56%, list=22%, signal=45%
HALLMARK_COAGULATION	58	0.6412982	2.79448476	1.00E-10	7.00E-10	2.86E-10	475	tags=48%, list=9%, signal=44%
HALLMARK_EPITHELIAL_MESENCHYMAL_TRANSITION	145	0.831003944	4.24287516	1.00E-10	7.00E-10	2.86E-10	696	tags=87%, list=13%, signal=77%
HALLMARK_HYPOXIA	107	0.545165592	2.66111549	1.00E-10	7.00E-10	2.86E-10	1335	tags=64%, list=26%, signal=49%
HALLMARK_INFLAMMATORY_RESPONSE	78	0.610277002	2.81831778	1.00E-10	7.00E-10	2.86E-10	1514	tags=79%, list=29%, signal=57%
HALLMARK_KRAS_SIGNALING_UP	76	0.582699307	2.68660839	1.00E-10	7.00E-10	2.86E-10	1160	tags=70%, list=22%, signal=55%
HALLMARK_TNFA_SIGNALING_VIA_NFKB	94	0.651074973	3.10466824	1.00E-10	7.00E-10	2.86E-10	1335	tags=77%, list=26%, signal=58%
HALLMARK_UV_RESPONSE_DN	72	0.562920793	2.56129974	3.37E-09	1.89E-08	7.70E-09	1377	tags=65%, list=27%, signal=49%
HALLMARK_ANGIOGENESIS	25	0.746713667	2.66701215	3.46E-09	1.89E-08	7.70E-09	1276	tags=96%, list=25%, signal=73%
HALLMARK_APOPTOSIS	61	0.578652589	2.54882173	8.04E-09	3.94E-08	1.61E-08	1222	tags=61%, list=24%, signal=47%
HALLMARK_P53_PATHWAY	70	0.514908841	2.33740157	9.57E-07	4.26E-06	1.74E-06	1057	tags=51%, list=20%, signal=42%
HALLMARK_MYOGENESIS	79	0.473066176	2.18638567	2.33E-06	9.53E-06	3.89E-06	834	tags=42%, list=16%, signal=36%
HALLMARK_COMPLEMENT	74	0.480478168	2.20863307	6.91E-06	2.60E-05	1.06E-05	516	tags=31%, list=10%, signal=28%
HALLMARK_TGF_BETA_SIGNALING	24	0.643250257	2.27794419	2.70E-05	9.45E-05	3.86E-05	1558	tags=83%, list=30%, signal=59%
HALLMARK_OXIDATIVE_PHOSPHORYLATION	79	-0.387568635	-2.1173314	3.00E-05	9.81E-05	4.00E-05	2479	tags=89%, list=48%, signal=47%
HALLMARK_IL2_STAT5_SIGNALING	81	0.428843859	1.99157207	5.63E-05	0.00017229	7.03E-05	1410	tags=58%, list=27%, signal=43%
HALLMARK_GLYCOLYSIS	95	0.377077318	1.80912909	0.00083293	0.0024008	0.00097992	1125	tags=45%, list=22%, signal=36%
HALLMARK_ALLOGRAFT_REJECTION	64	0.429298707	1.90738556	0.00105011	0.00285863	0.00116679	695	tags=31%, list=13%, signal=27%
HALLMARK_BILE_ACID_METABOLISM	36	-0.431770685	-1.919696	0.00137795	0.00355365	0.00145047	1163	tags=56%, list=22%, signal=43%
HALLMARK_MTORC1_SIGNALING	72	0.402164282	1.82985472	0.00179252	0.00439168	0.00179252	2171	tags=75%, list=42%, signal=44%
HALLMARK_IL6_JAK_STAT3_SIGNALING	28	0.525364719	1.91205585	0.00196303	0.00458039	0.00186955	710	tags=43%, list=14%, signal=37%
HALLMARK_INTERFERON_GAMMA_RESPONSE	42	0.445753158	1.8089003	0.00473197	0.01053938	0.00430179	1502	tags=57%, list=29%, signal=41%
HALLMARK_UNFOLDED_PROTEIN_RESPONSE	28	0.500401829	1.82120385	0.00503978	0.01073691	0.00438241	2328	tags=86%, list=45%, signal=48%
HALLMARK_MITOTIC_SPINDLE	54	0.412001785	1.75655231	0.00589651	0.01203871	0.00491376	2213	tags=76%, list=43%, signal=44%
HALLMARK_SPERMATOGENESIS	32	-0.389827532	-1.7100409	0.01100101	0.02156198	0.00880081	550	tags=28%, list=11%, signal=25%
HALLMARK_MYC_TARGETS_V1	53	0.395847569	1.68516254	0.01244932	0.02346218	0.0095764	2519	tags=85%, list=48%, signal=44%
HALLMARK_HEDGEHOG_SIGNALING	13	0.593744725	1.7813346	0.01396506	0.02534401	0.01034449	951	tags=69%, list=18%, signal=57%
HALLMARK_WNT_BETA_CATENIN_SIGNALING	18	0.518607589	1.69006	0.01549773	0.02712103	0.01106981	1219	tags=56%, list=23%, signal=43%
HALLMARK_FATTY_ACID_METABOLISM	75	-0.280834578	-1.4923884	0.02602354	0.04397081	0.01794727	1075	tags=36%, list=21%, signal=29%
HALLMARK_APICAL_SURFACE	16	0.522699585	1.66572501	0.0301659	0.04927097	0.0201106	1160	tags=62%, list=22%, signal=49%
